# Preliminary Findings on Cadmium Bioaccumulation and Photosynthesis in Rice (*Oryza sativa* L.) and Maize (*Zea mays* L.) Using Biochar Made from C3- and C4-Originated Straw

**DOI:** 10.3390/plants11111424

**Published:** 2022-05-27

**Authors:** Mohammad Ghorbani, Petr Konvalina, Reinhard W. Neugschwandtner, Marek Kopecký, Elnaz Amirahmadi, Jan Moudrý, Ladislav Menšík

**Affiliations:** 1 Department of Agroecosystems, Faculty of Agriculture and Technology, University of South Bohemia, Branišovská 1645/31A, 370 05 České Budějovice, Czech Republic; konvalina@zf.jcu.cz (P.K.); mkopecky@zf.jcu.cz (M.K.); amirae00@zf.jcu.cz (E.A.); jmoudry@zf.jcu.cz (J.M.J.); 2 Department of Crop Sciences, Institute of Agronomy, University of Natural Resources and Life Sciences Vienna, Konrad-Lorenz-Straße 24, 3430 Tulln, Austria; reinhard.neugschwandtner@boku.ac.at; 3 Division of Crop Management Systems, Crop Research Institute, 161 06 Prague, Czech Republic; ladislav.mensik@vurv.cz

**Keywords:** waste management, sustainable agriculture, nutrient storage, plant growth

## Abstract

Understanding the structural differences between feedstocks is critical for biochar effectiveness in plant growth. To examine the efficiency of biochars with unique physiological structures in a cadmium (Cd)-polluted soil, rice and maize as C3 and C4 plants, as well as biochar generated from their residues, defined as BC3 and BC4, were utilized. The experiment involved a control and a Cd-polluted soil (20 mg kg^−1^) without biochar application, and applications of each type of biochar (20 t ha^−1^) on Cd-polluted or unpolluted soil. In rice and maize fields, BC3 application led to the highest value of cation exchange capacity (CEC), with increases of 162% and 115%, respectively, over the control, while CEC increased by 110% and 71% with BC4 in the rice and maize field, respectively. As compared to the control, BC3 and BC4 dramatically enhanced the photosynthetic rate (Pn) of rice by 116% and 80%, respectively, and maize by 67% and 31%. BC3 and BC4 significantly decreased the Cd transfer coefficient in rice by 54% and 30% and in maize by 45% and 21%. Overall, BC3 is preferred over BC4 for establishing rice and maize in Cd-polluted soil, as it has a lower C/N ratio, a considerably higher surface area, and more notable alkaline features such as a higher CEC and nutrient storage.

## 1. Introduction

In order to improve photosynthesis and plant growth, the application of biochar has been widely conducted in cropping systems in recent years [1,2]. Several factors increase plant photosynthesis following biochar addition, such as the higher availability of water and nutrients (especially nitrogen), the higher cation exchange capacity (CEC) and porosity of the soil, more active microorganisms, as well as the immobilization of toxic metals [3,4]. The type of feedstock and its C/N ratio is an important factor as they directly affect the forming of the porous structure and absorbent characteristics of the biochar during the pyrolysis process [4,5]. There are contemporary reports on the effects of biochar derived from different types of feedstocks on photosynthesis and plant growth [5,6]. For example, wood residues, bamboo, and plant stems are relatively hard feedstocks with a C/N ratio > 50. Consequently, their degradability in the pyrolysis process is lower than that of feedstocks such as rice hull, rice straw, or wheat straw [7]. Thereby, the potential effectiveness of biochar derived from feedstocks with a higher C/N ratio will decrease in the root zone as the number of negative charges and functional groups on the biochar surface is lower [8].

On the other hand, two main types of photosynthetic structures (C3 and C4 plants) can have different responses to biochar. C4 plants have a more advanced mechanism for photosynthesis and stabilization of atmospheric carbon dioxide than C3 plants due to their physical structure [9]. C3 plants, such as rice or wheat, fix CO_2_ directly from the atmosphere and in mesophyll cells, while in C4 plants, such as maize or sugarcane, that process is conducted in specialized mesophyll and bundle sheath cells to participate in photosynthesis that is anatomically and biochemically distinct [10]. Typically, C4 plants have a 50% higher efficiency in photosynthesis rate (Pn) than C3 plants [9].

Regarding the role of soil metal toxicity in reducing plant photosynthesis efficiency, it should be noted that biochar addition to soil is also considered for preventing plants from heavy metal toxicity [11,12]. The concentration and toxicity of heavy metals have been widely considered in recent years due to the specific environmental problems they cause. The presence of heavy metals in soils, even in very low amounts, disrupts plant functions [12]. Cadmium (Cd) is one of the heavy metals that constitutes negative effects on ecosystems and food chain health [13]. Cd is entering into the soil through employing insecticides, irrigation with wastewater, and fertilization, as well as via metal retrieval industries. It has been widely shown that the presence of Cd in the soil causes reduction in plant growth, such as a reduction in root length and leaf number [14], and disturbances in the carbohydrate metabolism [11] and the photosynthetic system [3]. Prevention of chlorophyll synthesis is the main result of Cd bioaccumulation that is exhibited with biomass deficit and Pn reduction [15]. Cd stress furthermore alters stomatal movements, ion homeostasis, respiration in plants, and also prohibits the activities of enzymes [16]. Typically, the existing methods for reducing negative effects on plant growth are, however, costly and applicable to remediate small areas [12]. For example, enhanced phytoremediation of Pb-and Cd-contaminated agricultural soil with agricultural crops seemed not to be suitable in a reasonable time [17]. Furthermore, there is the risk of destruction of soil structure, disruption of soil biological activities, and environmental pollution [14]. Therefore, it is essential to provide a reliable and cheap method that minimizes contamination at low costs and is relatively fast without adverse effects on environmental health [16,18]. It has been widely shown that biochar can trap heavy metals in the soil and thus reduce their toxicity by relying on its unique characteristics such as high porosity and surface area [12,15].

It is apparent from earlier research that biochar can promote plant growth in heavy-metal-contaminated soils by immobilizing heavy metals. As a result, a main purpose of this study was to determine whether there is a difference in the effects of BC3 and BC4 on Cd mobilization, and if so, how large that difference is. Furthermore, because of their physiological variations, the response of rice and maize to Cd contamination can be interesting. However, a study comparing the responses of C3 and C4 plants to the addition of biochar has been overlooked thus far. Additionally, there has been insufficient research on the influence of homogeneous and heterogeneous biochar on the response of C3 and C4 plants. Homogeneous biochar is defined as the basic feedstock for biochar that is similar to treated plants (for example, rice straw biochar (BC3) applied to soil where rice is cultivated). Heterogeneous biochar is defined as the basic feedstock for biochar that differs from the treated plants (for example, rice straw biochar applied to soil where maize is cultivated). Therefore, we aimed to investigate the efficiency of rice and maize as C3 and C4 plants in response to the application biochars which are also produced from rice or maize straw. In this context, the hypothesis of this study was that biochars produced from C3 and C4 residues guarantee plant growth in Cd-contaminated soil. Additionally, it was expected that the application of two biochars would increase the photosynthesis rate of the plants due to its beneficial effects.

## 2. Results

### 2.1. Soil Properties

Biochar application in maize and rice fields significantly altered all soil properties (*p* < 0.01) (Table 1). The highest pH was found in BC3 + Cd-treated soil on both maize and rice fields, with a 2.51 and 3.01-unit increase in the rice field and maize field, respectively, as compared to the control. In rice fields, BC3 caused the greatest CEC value of 22.3 cmol^(+)^ kg^−1^ (162% increase compared to the control). With a value of 17.9 cmol^(+)^ kg^−1^, BC4 application had the second highest CEC (110% increase compared to the control). BC3 and BC4 generated a considerable increase in CEC in the maize field by 115 and 71%, respectively, when compared to control. In the rice field, BC3 enhanced the value of exchangeable K^+^, Ca^2+^, and Mg^2+^ by 484, 310, and 218%, respectively, compared to the control, and in the maize field, by 196, 127, and 195%, respectively. Both BC3 and BC4 treatments significantly increased OC in the rice field, but there was no significant difference between BC3 and BC4. BC3 has the highest concentration of OC, at 2.13%. Additionally, in the maize field, the OC was highest with 2.23% after BC3 application.

The highest total N levels in the rice field were associated with BC3 and BC4 application, with values of 168 and 136 mg kg^−1^, respectively. The amount of N decreased dramatically in the Cd-polluted soil when compared to the control (69%). The concentrations of available P and K were also significantly raised when BC3 and BC4 treatments were used. The highest availability of P and K was related to BC3 with an increase of 141% and 109%, respectively, compared to the control. BC3 + Cd and BC4 + Cd showed a significant decrease in P concentration compared to their corresponding treatments (BC3 and BC4) with values of 54.3 and 47.4 mg kg^−1^. Similar to P, values of K in BC3 + Cd and BC4 + Cd treatments were significantly lower than BC3 and BC4 with values of 142 and 138 mg kg^−1^, respectively. In addition, the Cd-polluted treatment resulted in a substantial drop in both soil parameters (P and K) when compared to the control (with a 28% and 27% decrease for P and K, respectively).

In the maize field, the BC3 and BC4 treatments resulted in substantial differences in N, P, and K when compared to the control, as well as a significant difference between BC3 and BC4. The highest values of N, P and K were related to BC3 with a 92%, 83%, and 33% increase compared to the control, respectively. BC3 + Cd revealed a significant decrease compared to its corresponding treatment (BC3) with a reduction of 27% in N, 18% in P, and 9% in K. In addition, as compared to the control, the Cd-polluted treatment generated a significant difference in N, P, and K, with a 38%, 45%, and 28% decrease, respectively.

### 2.2. Plant Growth and Photosynthesis Rate

**Figure 1:**
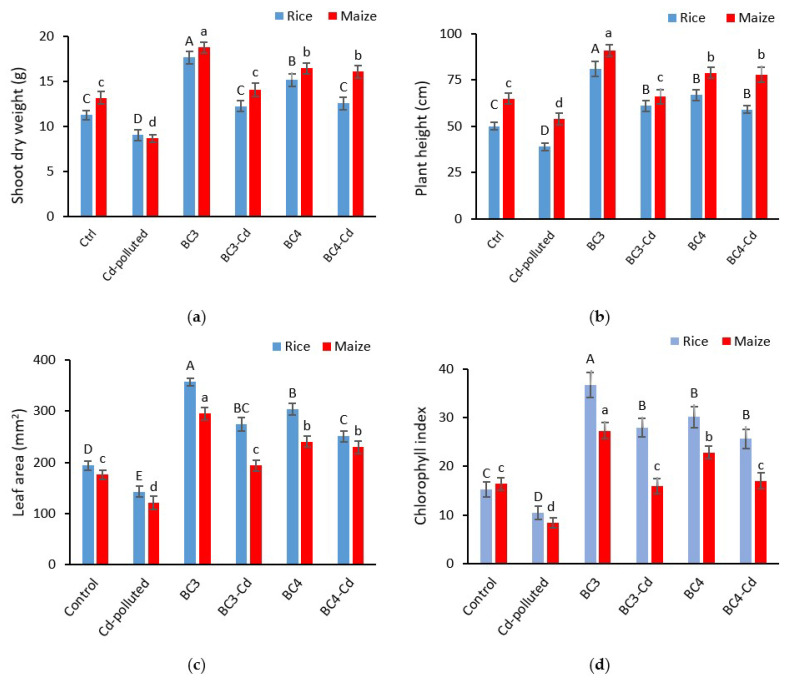
Influence of biochar and Cd treatments on shoot weight (**a**), plant height (**b**), leaf area (**c**), and chlorophyll index (**d**) in rice and maize fields as representatives of C3 and C4 plants, respectively. Significant differences of means are shown by different uppercase letters for rice and lowercase letters for maize (*p* < 0.01). The values are means ± SD from three replicates (*n* = 3). BC3: rice biochar, BC4: maize biochar, BC3 + Cd: rice biochar + cadmium, and BC4 + Cd: maize biochar + cadmium.

In rice and maize fields, biochar application significantly boosted plant growth metrics (*p* < 0.01) (Figure 1). In the rice field, BC3 and BC4 induced a considerable increase in shoot dry weight of 57% and 34%, respectively, and 42% and 25% in the maize field. The shoot dry weight of rice and maize was reduced by 19% and 34% in the Cd-polluted treatment, respectively, as compared to the control (Figure 1a).

The addition of BC3 boosted the rice height to 81 cm, resulting in a 62 percent increase above the control, while the plant height after BC4 application was 67 cm. Moreover, the maize plant height was the highest with BC3, with a value of 91 cm and a 40% increase over control. Additionally, with a value of 79 cm and a 21% increase above the control, BC4 produced the second greatest plant height. Cd-polluted treatment resulted in a significant decrease in either rice and maize plants with 22% and 17% decrease compared to the control, respectively (Figure 1b).

In the rice field, BC3 and BC4 generated a considerable increase in leaf area of 84% and 57%, respectively, and 68% and 37% in the maize field. The Cd-polluted treatment caused a significant decrease in leaf area of rice and maize by 26% and 31% compared to control (Figure 1c).

BC3 and BC4 considerably enhanced the chlorophyll content in the rice field by 140% and 97%, respectively, and the value of chlorophyll content was significantly greater with BC3 than with BC4. The chlorophyll content significantly decreased in the Cd-polluted treatment by 32% compared to the control. BC3 resulted in a considerable increase in chlorophyll content of 67% in the maize field, and BC4 came in second with a 39% gain over the control. Both BC3 + Cd and BC4 + Cd showed a significant decrease compared to their corresponding treatments (BC3 and BC4) (Figure 1d).

**Figure 2:**
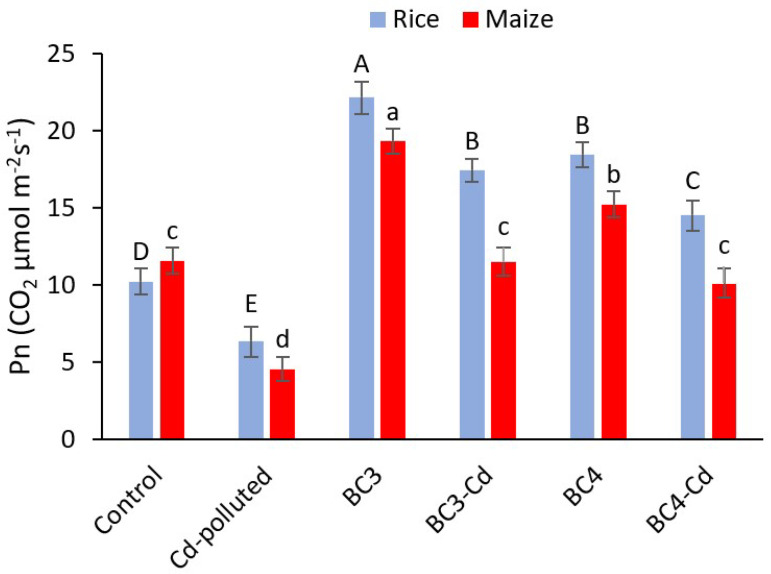
Effects of biochar and cadmium treatments on photosynthesis rate (Pn) in rice and maize field as representatives of C3 and C4 plants, respectively (means ± standard error). Significant differences of means are shown by different uppercase letters for rice and lowercase letters for maize (*p* < 0.01). The values are means ± SD from three replicates (*n* = 3). BC3: rice biochar, BC4: maize biochar, BC3 + Cd: rice biochar + cadmium, and BC4 + Cd: maize biochar + cadmium.

Rice and maize photosynthetic rates (Pn) were significantly affected by the two types of biochar (*p* < 0.01) (Figure 2). BC3 had the highest value of Pn of rice, with a 116% increase above the control. The second highest value was related to BC4 with an 80% increase compared to the control. Pn was significantly greater with BC3 (22.12 CO_2_ µmol m^−2^ s^−1^) than with BC4 (18.43 CO_2_ µmol m^−2^ s^−1^). BC3 + Cd and BC4+Cd treatments resulted in a significant decrease in Pn compared to their corresponding treatments (BC3 and BC4) with values of 17.4 and 14.5 mg kg^−1^ CO_2_ µmol m^−2^ s^−1^. Additionally, the Cd-polluted treatment showed a significant difference in Pn with a 38% decrease compared to the control. In addition, in maize, the Pn was significantly higher with BC3 than with BC4. The highest Pn value was observed with BC3 with a 67% increase compared to the control. BC4 application resulted in the second highest Pn value with a 31% increase compared to the control. The Pn in the BC3 + Cd and BC4 + Cd treatments was significantly lower compared to their corresponding treatments (BC3 and BC4). Additionally, the Pn in the Cd-polluted treatment exhibited a substantial difference, with a 61% drop compared to the control.

**Figure 3:**
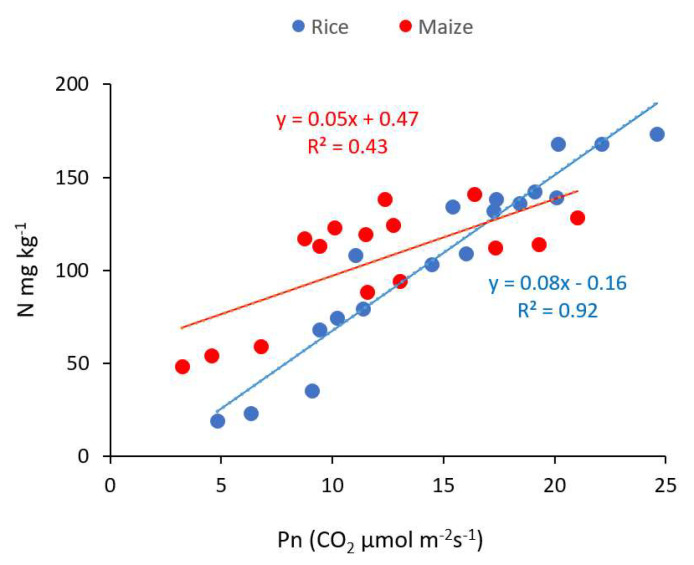
Effects of biochar and cadmium treatments on photosynthesis rate (Pn) in rice and maize field as representatives of C3 and C4 plants, respectively (means ± standard error). Significant differences of means are shown by different capital letters for rice and lowercase letters for maize (*p* < 0.01).

The Pn increased with the N concentration in the soil, with a stronger increase in the maize field. A positive coefficient of determination between N and Pn was obtained in the rice field (R^2^ = 0.92) and the maize field (R^2^ = 0.43) (Figure 3).

### 2.3. Cd Bioaccumulation Factor and Transfer Coefficient

**Figure 4:**
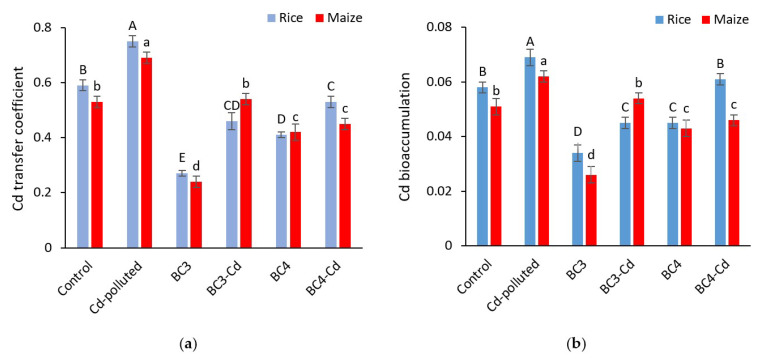
Effects of biochar and cadmium treatments on Cd bioaccumulation (**a**) and transfer coefficient (**b**) in rice and maize fields as representatives of C3 and C4 plants, respectively (means ± standard error). Significant differences of means are shown by different uppercase letters for rice and lowercase letters for maize (*p* < 0.01). The values are means ± SD from three replicates (*n* = 3). BC3: rice biochar, BC4: maize biochar, BC3 + Cd: rice biochar + cadmium, and BC4 + Cd: maize biochar + cadmium.

The Cd transfer coefficient was considerably altered by biochar application (*p* < 0.01) (Figure 4a). BC3 and BC4 significantly reduced the Cd transfer coefficient in rice by 54% and 30% decrease compared to the control, respectively. With a value of 0.75, the highest Cd transfer coefficient was related to Cd-polluted treatment (27% increase compared to control). BC3 treatment resulted in the lowest Cd transfer coefficient in maize, with a 45% reduction compared to the control and BC4 had the second lowest Cd transfer coefficient, with a reduction of 21% when compared to control. Similar to rice, the maximum Cd transfer coefficient was found from Cd-polluted treatment, with a 30 percent increase above the control.

Rice and maize bioaccumulation of Cd was significantly reduced after biochar treatment (*p* < 0.01) (Figure 4b). Both types of biochar reduced Cd bioaccumulation in rice, although BC3 had the lowest Cd bioaccumulation, with a 41% reduction when compared to the control. BC3, which has a lower bioaccumulation than BC4, differed significantly from BC4. In addition, BC3 had the lowest Cd bioaccumulation in maize, with a 49% reduction compared to the control. The Cd-polluted treatment resulted in the largest Cd bioaccumulation in both plants, with increases of 19 and 22%, respectively, as compared to the control.

## 3. Discussion

The application of biochar positively increased pH, CEC, and exchangeable cations in the experimental fields which had basically an acidic nature. As the research site is located in a tropical region and thereby generally influenced by high precipitation and temperature, it is exposed to the loss of basic cations [19]. Biochars produced from crop residues are here able to prevent re-acidification of acidic soils by boosting the soil pH-buffering capacity [20]. Large surface area of biochar with considerable functional groups (for example, carboxylic and phenolic groups) is the key factor to increasing the pH of biochar [5]. Furthermore, it contains different mineral nutrients in its ash including basic cations (K^+^, Ca^2+^, and Mg^2+^). Therefore, biochar can potentially buffer soil pH by adding basic cations and consuming protons with negatively charged functional groups [21]. BC3 had a higher ash content compared to BC4 and consequently a higher CEC and higher values for basic cations, surface area, and pH. This is the main reason why the application of BC3 considerably better ameliorated the acidic soils than BC4 in both fields. Cd–biochar treatments significantly increased pH compared to the control. These results are in line with some previous studies which reported that using CaO-containing biochar in Cd-polluted treatment caused an increase in soil pH due to dissolving CaO and release of OH-ions into the soil solution [16,20]. In addition, the availability of N, P, and K also sharply increased in BC3 and BC4 treatments. This can be explained by the more porous structure and high surface area in BC3 than BC4. During pyrolysis, volatile compounds are released in the form of gases, which can generate a particularly porous honeycomb structure as well as increase the surface area of biochar. As a consequence, water and nutrient storage in the soil will be improved by applying biochar. In contrast, treatments that contain Cd showed negative effects on OM and N, P, and K availability. This means that Cd challenges for absorption of several mineral nutrients with the same chemical properties such as Ca^2+^ and Mg^2+^ in the root zone, thereby causing a mineral deficiency [12]. Moreover, the accessibility of nitrates, phosphate potash, and sulfates in soil, which do not have the same chemical characteristics as Cd, is prevented by the Cd bioaccumulation. A decrease in macronutrients in the tissue due to high concentrations of Cd has been reported in previous pieces of literature [16,20].

The application of BC3 to both plants significantly reduced the bioaccumulation and the transformation of Cd compared to BC4. Typically, biochar application in agricultural soil induces important surface characteristics changes due to a consequence of biochemical interplays, which are correlated with improving the adsorption behavior of cations [7]. Biochar has many functional groups such as carboxylate and hydroxyl groups [15] and has the potential for electrostatic interaction [22], ion exchange [21], and a strong surface complex with heavy metals [23]. Therefore, those intrinsic adsorbent properties in biochar play an important role in stabilizing Cd and increasing the concentration of non-absorbable Cd in the soil [21]. Thus, a decrease in Cd uptake in plants is expected [12] due to the surface characteristics of biochar [21,24]. This is the main reason why BC3 application reduced bioaccumulation of Cd in rice and maize by 41% and 49%, while the reduction with BC4 in rice and maize fields was at 22% and 16% compared to control. Next to the higher surface area and CEC, BC3 also resulted in an increase in the chlorophyll contents and photosynthesis activity compared to BC4, further supporting the higher suitability of BC3. It has been reported that biochar from rice residues caused an immobilization of Cd by 97% in the soil [15], while biochar derived from wood residues, bamboo, maize stem, and nutshells stabilized Cd less by 60% [25,26]. This can be related to the lower C/N ratio of rice husks compared to that of other feedstocks. A high C/N ratio of the feedstock causes the incomplete formation of adsorption properties at the biochar surface during the pyrolysis process and thus reduces Cd immobilization in soil [27]. The lower C/N ratio of BC3 than that of BC4 can confirm this hypothesis. BC3 also reduced the Cd transformation towards grains significantly more than BC4 which was the desired result. Moreover, it has been proven that rice husk biochar incorporation in soil provides silicon (Si) and other nutrients and improves their mobility in soils under Cd stress [15]. In fact, the addition of biochar from rice residues significantly contributes to nutrient cycling in the soil–plant system and mitigates Cd translocation and its deleterious effect on rice growth [28].

The addition of BC3 specifically increases the growth of rice and maize. The positive growth responses were attributed directly by biochar-supplied nutrients [3]. In this study, biochar BC3 provided more available nutrients (Ca, Mg, K, and N) and higher EC than BC4. The EC value represents the value of water-soluble nutrients [22]. Consequently, the improved nutrient contents in the soil such as available Ca, Mg, and K could enhance nutrient uptake and benefit plant growth [29]. Smaller shoot dry weight in Cd-contaminated treatment was probably due to the toxic Cd concentrations in plants [12], which lead to a disturbance in the metabolic processes [30]. When no Cd treatment was applied, BC3 could add sufficient nutrients into the soil. This is because of the high surface area of biochar produced from rice husk that can ameliorate Cd toxicity by stabilization of it in soil [21,24]. Increasing the plant height and leaf area resulted in an increase in the shoot dry weight due to preventing Cd uptake by plant roots. The application of biochar prevented the disruptive effect of Cd and increased the amount of chlorophyll in the leaf by decreasing the Cd bioaccumulation in the plant and the transfer coefficient.

Cd concentration in plants caused interference with the chlorophyll synthesis process, disrupting it [3]. It is also possible that biochar improves the photosynthesis of hydrocarbon materials and increases the production of biomass by increasing the amount of chlorophyll content [31].

The photosynthesis rate of both plants significantly increased with application of biochar, especially of BC3. This increase could be considered as a consequence of improving high leaf area and chlorophyll content following BC3 application. BC3 significantly increased Pn in rice and maize by 116% and 66% compared to the control, while increases in the Pn of rice and maize after the application of BC4 were at 80% and 31% compared to the control. The better performance of BC3 is related to its high surface area and nutrient storage than BC4. In biochars analyzing, the amount of N and NO_3_^−^-N input with BC3 (218 kg N ha^−1^ and 1.96 kg NO_3_^−^-N ha^−1^) was consequently higher than that from BC4 (90 kg N ha^−1^ and 1.32 kg NO_3_^−^-N more). There was a significant positive correlation between N in the soil and the Pn of rice. It has been proven that with an increase in total N in the soil and thereafter an increase in the N concentration in the plant, both a higher leaf area and chlorophyll content can be expected [3,32]. Typically, the presence of the rubisco enzyme as well as the N concentration are higher in C3 than in C4 plants [30]. It shows a larger N store in photosynthetic enzymes and a higher N demand of C3 plants than of C4 plants. Hence, the enhancement in the N uptake by the plant and the prevention of N leaching from the soil due to applying biochar [3] are helpful for boosting the photosynthesis and plant growth of C3 plants compared to C4 plants. Previous studies have shown that C4 plants tend to have lower water potential shortage and stomatal conductance than C3 plants [32]. Hence, C4 plants cannot as much use the advantages of biochar application as C3 plants, which is supported by our results of the higher biochar-induced increase in Pn for rice but lower increases in this parameter for maize.

## 4. Materials and Methods

### 4.1. Soil, Plant, and Biochar Preparation

The study was conducted in 2020 at the Agricultural Technology and Natural Resources Development Center (37°11′2.5″ N 49°39′36.6″ E) in Gilan, Iran. Some environmental parameters of study area are presented in Table 2. Rice and maize were selected as representatives of C3 and C4 plants, respectively, for the evaluation of their response to biochar application to Cd-contaminated soil. Two types of biochar were also produced from residues of the selected plants (rice or maize straw), called hereafter BC3 and BC4, by a rotary furnace. After about two hours of slow pyrolysis at 450 °C, cooking was completed by sprinkling water on the biochar (see Ghorbani et al., 2021 for details). To achieve the Cd-contaminated soil, cadmium nitrate (Cd(NO_3_)_2_) solution at 20 mg kg^−1^ soil (equal to 4.48 g per m^2^ soil with a depth of 15 cm and a bulk density of 1.68 g m^−3^) was spiked to the soil one week before planting. At the same time, biochar types were manually spread on the research field at a rate of 20 t ha^−1^ and homogeneously mixed by tractor plowing into the topsoil (25 cm). The experiment involved a control and a Cd-polluted soil without biochar application, and applications of each type of biochar on Cd-polluted or unpolluted soil. Consequently, six treatments were performed and named: control, Cd-polluted, BC3, BC3 + Cd, BC4, and BC4 + Cd.

Rice (*Oryza sativa* L.) cv. Hashemi and maize (*Zea mays* L.) cv. Single Cross 704 were grown in April 2020 in two separate fields but close to each other on the same soil with clay texture. Therefore, for each field, 18 plots including 6 treatments and 3 replications were performed (36 plots in total for both fields). Plot size was 20 m^2^ (4 m × 5 m).

### 4.2. Soil and Biochar Analysis

According to the USDA soil taxonomy system the experimented soils were calcified in anthrosols and some soil properties were analyzed before and at the end of experiment by following methods: soil pH and electrical conductivity (EC) in a 1:1 (*w:v*) soil to water ratio; soil texture by hydrometer (Beretta et al., 2014); organic carbon (OC) by wet oxidation [33]; total nitrogen (N) by Kjeldahl [34]; and CEC by ammonium acetate extraction (Tournassat et al., 2004). Exchangeable K^+^, Ca^2+^, and Mg^2+^ were analyzed using a 5:50 ratio of soil:ammonium acetate (NH_4_OA_c_)-buffered solution at pH 7, in which the basic cations adsorbed in soil were replaced by NH_4_^+^ ions [35] and measured by spectroscope (ICP-OES, PerkinElmer). The atomic absorption method was used for Cd measuring [36] (Table 3).

Biochar properties were measured as following methods: pH and EC by 1:20 (*w:v*) biochar to water ratio [37]; carbon (C), hydrogen (H), and nitrogen (N) by the elemental analyzer (Perkin Elmer 2400 II); CEC and exchangeable cations by ammonium acetate method [38]; and specific surface area by the Brunner, Emmett, and Teller (BET) procedure [39] (Table 3).

### 4.3. Sampling and Measurements

According to [40], the photosynthetic rate was analyzed in five selected plants per plot in week 13 after the start of the experiment using a portable photosynthesis device (Li-6400XT, NE, USA). The chlorophyll index was determined by a 508 SPAD chlorophyll meter and the leaf area was measured with the Delta-T (Divises Ltd., Hatfield, UK). After four months (at the harvest time), one square meter of plants was harvested diagonally from each plot and the height of plants was measured by a T-ruler. Harvested plants, after washing, were placed for 48 h in a 60 °C oven. Plant samples (shoots and roots) were powdered with a laboratory mill Cd analysis.

The transfer coefficient of Cd and the bioaccumulation factor of Cd were calculated by Equations (1) and (2) [36] as follows:

This is example 1 of an equation:

Transfer coefficient = (mg of Cd in the shoot)/(mg of Cd in the root)(1)

Bioaccumulation factor = (mg of Cd in the soil)/(mg of Cd in the plant)(2)

### 4.4. Data Analysis

The statistical analysis of the effects of two types of biochar and Cd pollution on plant growth were performed in two-factorial arrangement in a completely randomized design with three replicates. The triplicate data of selected soil properties and growth characteristics were subjected to analysis using the 2-way ANOVA test, conducted by SPSS 23.0 software. Treatment means were separated using the least significant difference test. Least-square means were used to test for significant differences among the treatments at *p* < 0.01. Linear regression analysis was performed to investigate the relationships among photosynthesis rate (Pn) and total soil nitrogen (N) using Excel 2018.

## 5. Conclusions

Rice and maize are considered staple crops for their uses, including food for humans and feed for animals. However, soil acidity and Cd pollution are constraints for rice and maize cultivation. In the current study, biochars derived from rice or maize residues have different chemical properties and absorbent characteristics. Biochar produced from rice straw was more efficient compared to biochar for improving photosynthesis characteristics of both rice and maize in acidic soil and for mitigating Cd bioaccumulation. In fact, it can be said that the mere use of biochar does not guarantee an improvement in plant growth characteristics under stressful conditions. In other words, differentiation of feedstocks in terms of the ratio of C/N and the degree of degradability is a determining factor in the establishment of the plant in the contaminated environment, the availability of an adequate nutritional source for the plant, and plant growth. Therefore, with regard to the wide range of agricultural products, the efficient use of rice straw biochar can be a step forward in the proper management of agricultural residues. These findings can be regarded as preliminary, and future long-term studies may shed light on additional facets of the issue.

## Figures and Tables

**Table 1:** Chemical properties of soil as affected by treatments after four months (end of experiment).

Treatments	pH	CEC (cmol^(+)^ kg^−1^)	Exchangeable Cations (cmol^(+)^ kg^−1^)			OC (%)	N (mg kg^−1^)	P (mg kg^−1^)	K (mg kg^−1^)
			K	Ca	Mg				
**Rice field**									
Control	5.01 ^d^	8.49 ^c^	2.24 ^d^	4.07 ^d^	3.05 ^d^	1.06 ^b^	74 ^c^	28.3 ^c^	119 ^c^
Cd-polluted	5.12 ^d^	5.63 ^d^	0.65 ^e^	0.94 ^e^	1.25 ^e^	0.97 ^b^	23 ^d^	20.4 ^d^	87 ^d^
BC3	6.44 ^bc^	22.3 ^a^	13.1 ^a^	16.7 ^a^	9.71 ^a^	2.13 ^a^	168 ^a^	68.1 ^a^	178 ^a^
BC3 + Cd	7.52 ^a^	15.1 ^b^	6.81 ^b^	8.21 ^b^	7.22 ^b^	1.83 ^a^	138 ^ab^	54.3 ^b^	142 ^b^
BC4	6.09 ^c^	17.9 ^b^	5.32 ^bc^	6.83 ^bc^	8.24 ^b^	1.94 ^a^	136 ^ab^	59.2 ^a^	167 ^a^
BC4 + Cd	6.68 ^b^	9.21 ^c^	4.14 ^c^	5.49 ^c^	5.25 ^c^	1.71 ^a^	103 ^b^	47.4 ^b^	138 ^b^
**Rice field**									
Control	4.97 ^d^	9.02 ^c^	2.19 ^d^	4.38 ^d^	3.49 ^d^	1.12 ^c^	88 ^c^	36.8 ^c^	144 ^c^
Cd-polluted	5.04 ^d^	6.24 ^d^	0.17 ^e^	1.25 ^e^	0.98 ^e^	1.01 ^c^	54 ^d^	20.2 ^d^	103 ^d^
BC3	6.67 ^bc^	19.4 ^a^	6.48 ^a^	9.94 ^a^	10.3 ^a^	2.23 ^a^	169 ^a^	67.3 ^a^	191 ^a^
BC3 + Cd	7.98 ^a^	17.2 ^b^	5.13 ^b^	6.34 ^b^	7.51 ^b^	2.03 ^a^	123 ^b^	55.2 ^b^	173 ^b^
BC4	6.16 ^c^	15.5 ^b^	3.39 ^c^	5.96 ^bc^	7.04 ^b^	1.87 ^b^	121 ^b^	54.9 ^b^	179 ^b^
BC4 + Cd	7.04 ^b^	10.3 ^c^	3.24 ^c^	5.22 ^c^	5.14 ^c^	1.67 ^b^	119 ^b^	53.3 ^b^	176 ^b^

EC: electrical conductivity, CEC: cation exchange capacity, C: carbon, H: hydrogen, N: nitrogen, O: oxygen. BC3 and BC4: biochar produced from rice and maize straw, respectively. In each column different lowercase letters show significant differences of means (*p* < 0.01). The values are means from three replicates (*n* = 3). BC3: rice biochar, BC4: maize biochar, BC3 + Cd: rice biochar + cadmium, and BC4 + Cd: maize biochar + cadmium.

**Table 2:** Environmental description of the study area.

Site Property	Description
General climate	Humid temperate continental monsoon climate
Average annual air temperature (°C)	17.2
Frost-free period (day)	250
Average annual precipitation (mm)	1359
Duration of sunshine (h year^-1^)	1938.3
Parent material	Fluvial alluvium
Clay minerals	Mainly mica and montmorillonite
Soil classification (WRB)	Hydragric anthrosol
Soil tillage system	Rotation

**Table 3:** Selected properties of soil and biochar.

Property	Rice Field	Maize Field	BC3	BC4
pH	5.72	5.68	8.97	7.95
EC (dS m^−1^)	0.21	0.18	0.63	0.52
CEC (cmolc kg^−1^)	7.82	8.95	45.7	19.4
Specific surface area (m^2^ g^−1^)	-	-	92.3	36.4
Organic C (%)	1.13	1.06	54.6	46.1
H (%)	-	-	2.21	3.82
O (%)	-	-	18.2	25.9
N (%)	0.67	0.56	1.09	0.64
C/N ratio	-	-	50	72
NO_3_^-^-N (g kg^−1^)	0.028	0.019	0.098	0.032
Exchangeable K (cmol^(+)^ kg^−1^)	2.28	2.21	25.53	11.38
Exchangeable Ca (cmol^(+)^ kg^−1^)	3.92	4.12	30.34	12.21
Exchangeable Mg (cmol^(+)^ kg^−1^)	3.21	3.41	22.87	8.08
Ash content (%)	-	-	38.4	19.3
Sand (%)	8.6	9.5	-	-
Silt (%)	31.6	35.2	-	-
Clay (%)	59.8	55.3	-	-
Texture	Clay	Clay	-	-

EC: electrical conductivity, CEC: cation exchange capacity, C: carbon, H: hydrogen, N: nitrogen, O: oxygen. BC3 and BC4: biochar produced from rice and maize straw, respectively.

## Data Availability

Not applicable.
